# Hypertension, the changing pattern of drug usage

**Published:** 2009-02

**Authors:** Opie Lionel H

**Affiliations:** Hatter Institute for Cardiovascular Research, Department of Medicine, Chris Barnard Building, Faculty of Health Sciences, University of Cape Town, Observatory

## Abstract

**Summary:**

Gradually the pattern of use of antihypertensive drug agents has changed, from prime use of diuretics and beta-blockers, to preference for the inhibitors of the renin-angiotensin system and the calcium channel blockers. In assessing the value of potentially conflicting evidence, attention should be paid to the hierarchy of evidence, which works its way up through 10 steps from isolated case reports to integrated knowledge.

## Summary

In 1979, Prof Willem Lubbe, then director of the Hypertension Clinic at Groote Schuur Hospital, left for New Zealand, leaving me to inherit his first-class hypertension clinic. What have the major changes been in the last thirty years since then? I will single out four. First, there is now widespread and increasing recognition that hypertension is a crucial risk factor, not only for coronary heart disease (CHD) and stroke, but also for renal damage, especially in the presence of diabetes. Hypertension, once a backstage player in the cardiovascular arena, has therefore moved to centre stage. The second change relates to the ideal blood pressure (BP) level, which has consistently dropped from simply being below 160/95 mmHg to current levels of about 115/75 mmHg.

**Fig. 1.**
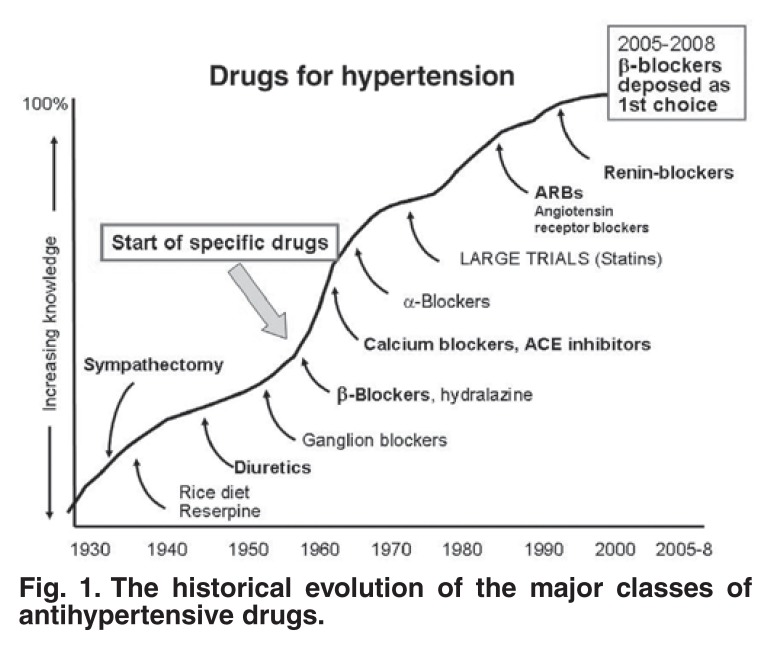
The historical evolution of the major classes of antihypertensive drugs.

The third major change, and the change that I wish to concentrate on, is that drug therapy for hypertension has become almost free of side effects. The simplicity of drug therapy for most patients and the emphasis on the benefits of lifestyle changes have made hypertension often one of the easiest of the cardiovascular risk factors to treat. Thirty years ago, most of the drugs that we presently have to treat hypertension were already defined and tested, albeit not all with good outcome studies (Fig. 1). The fourth change is that over the years we have seen and are still experiencing major changes in the patterns of drug usage for hypertension.

## Diuretics and beta-blockers

For many years these drugs have been the mainstay of the therapy of hypertension. The story of their prolonged but now passing glory starts in 1963, when I was working with Prof AJ Brink, Editor-in-Chief of this Journal, under whom I had the honour of working in my early postgraduate career. In the *Lancet* in 1963, Cranston and colleagues from the Regius Department of Medicine at Oxford University, presented the first human dose-response study with the diuretics.^1^ The famous statement made in that article was: ‘Little benefit is to be derived from using large doses of oral diuretics to reduce blood pressure’. Thus the issue of an optimal dose was launched. I also learnt that the mechanism of action of diuretics was not well understood, and that high doses could cause diabetes and hypokalaemia. Even today, these remain as the chief drawbacks of diuretic therapy with one (hypokalaemia) seeming to cause the other (diabetes).

## Evolution of beta-blockers

Beta-blockers were the first group of drugs specifically created to fit into a receptor. The creator was Sir James Black, who was later awarded a Nobel Prize for advances in medicine. He was passionate about creating a drug that opposed the angina-provoking effects of catecholamines. Logically, as beta-receptor over-activity promoted tachycardia and hypertension, it was also possible that the new beta-blocking drugs could reduce hypertension, as was elegantly shown by Brian Prichard in 1964.^2^ Therefore, not surprisingly, for many years the standard first-line therapy for hypertension was a combination of the two oldest tested agents, namely diuretics and beta-blockers (I am excluding ganglion-blocking drugs and reserpine because they could cause severe side effects). The ACE inhibitors and calcium channel blockers were next in the line of evolution.

## The beta-blocker counter-revolution

**Fig. 2.**
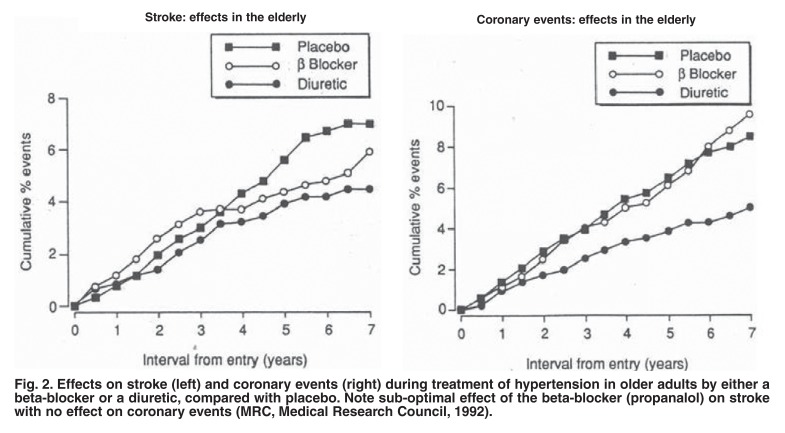
Effects on stroke (left) and coronary events (right) during treatment of hypertension in older adults by either a beta-blocker or a diuretic, compared with placebo. Note sub-optimal effect of the beta-blocker (propanalol) on stroke with no effect on coronary events (MRC, Medical Research Council, 1992).

Then, in 1992, one of the first large, well-designed, placebo-controlled outcome studies in elderly British patients came out.^3^ Using propranolol as the beta-blocker compared with a diuretic, there was clearly little benefit against stroke and none on coronary events (Fig. 2). With time, an increasingly strong resistance developed against the prime use of beta-blockers, initially led by Messerli,^4^ who used the technique of meta-analysis to group together the available studies to show that in elderly patients, beta-blockers gave worse outcomes than did diuretics.

Another argument developed against beta-blockers, based on clinical observations. As time went on in the Hypertension Clinic of Groote Schuur Hospital, I could not help noticing how many patients on combined beta-blocker and diuretic therapy were developing diabetes. Admittedly, they were generally overweight, which would have promoted the process, but there were increasing literature indications that beta-blockers were at fault. In a modified meta-analysis in 2004 (Table 1), it was possible to show that the ‘old’ antihypertensives, namely the beta-blockers and diuretics, produced more new diabetes than did the ‘new’ antihypertensives, such as the ACE inhibitors and the calcium channel blockers.^5^

**Table 1:** Beta-Blocker (BB) Comparisons In Antihypertensive Trials: Relative Risks

	*Total mortality*	*CHD*	*Stroke*	*CV mortality*
BB vs placebo	0.99 NS	0.93 NS	0.80 (0.66–0.96)	0.93 NS
BB vs diuretic	1.04 NS	1.12 NS	1.17 NS	1.09 NS
BB vs CCB	1.06 (1.00–1.14)	1.05 NS	1.24 (1.11–1.40)	1.15 NS
BB vs RAS blockers	1.08 NS	0.90 NS	1.30 (1.11–1.53)	1.09 NS

CHD, coronary heart disease; CV, cardiovascular. Data from Opie and Schall.^5^

The real push against beta-blockers developed when Lindholm, from Sweden, published a major opinion-breaking article in the *Lancet* in 2005, showing that beta-blockers as a group, but chiefly atenolol, were only half as effective as they should have been in preventing stroke.^6^ The proposed mechanism was that beta-blockers, by reducing the heart rate, alter the aortic wave pattern in such a way that the systolic aortic pressure, which reaches the brain, heart and kidneys, decreases less than the brachial pressure.^7^

To put it differently, compared with other antihypertensives taken as a group, it could be said that beta-blockers relatively promoted stroke. Very shortly after that our ‘Southern Cape Universities’ meta-analysis, led by Hazel Bradley, came out. We were able to give information on how beta-blockers compared with other groups of agents. Versus placebo, beta-blockers as a group did not decrease coronary heart disease or total mortality, but modesty decreased stroke by 20%.^8^ We also submitted as a *Cochrane Review*, which was accepted,^9^ a strong plea for a revision of therapeutic habits in an editorial in this Journal.^10^

The result of all these publications was that the British Hypertension Society demoted beta-blockers to third- or fourth-line therapy for hypertension and the South African Hypertension Society followed suit. Therefore I was very surprised when in 2007 the European recommendations appeared and suggested that beta-blockers were among possible first-line antihypertensive drugs. Accordingly, I wrote an article provocatively titled ‘Beta-blockers should not be among first-line therapy for hypertension’ and this appeared in the *Journal of Hypertension*,^11^ with a counter-balancing set of arguments by two of the major authors of the European Recommendations, namely Georgio Mancia and Alberto Zanchetti.^12^ I had actually given seven major reasons why beta-blockers should not be used as first-line therapy (Table 2).

**Table 2:** Reasons Why Beta-Blockade Should Not Be Among Several Choices For Initial Therapy Of Hypertension, Unless Reasons Are Compelling; Note, No Data On Vasodilatory Beta-Blockers

*Problem related to beta-blocker antihypertensive therapy*	
1	Diabetogenic potential no longer in doubt
2	ASCOT BP-lowering arm showed outcome inferiority of therapy initiated with atenolol versus that initiated with CCB, amlodipine (including mortality disadvantage)
3	When compared with other antihypertensives, stroke is reduced less than expected, probably due to lesser reduction in central aortic pressure
4	Lack of regression of left ventricular hypertrophy
5	Male sexual dysfunction
6	Case for cardioprotection overstated; see ref 13
7	Specific disadvantages in metabolic syndrome and obesity (weight gain)

However, probably the really lethal blow to beta-blockers came with the recent article by Messerli’s group.^13^ These authors found nine controlled trials evaluating beta-blockers for hypertension that had reported heart rate data. Unexpectedly, a lower heart rate at the end of the study in the beta-blocker group was associated with a greater risk for all-cause mortality, cardiovascular mortality, myocardial infarction and heart failure. This led the doyen of hypertension, Norman Kaplan (with whom I have had the honour to write the chapter on hypertension for six consecutive editions of *Drugs for the Heart*, starting in 1985) to conclude as follows: ‘With this addition to the evidence, betablockers will surely remain as indicated for heart failure, for after myocardial infarction and for tachyarrhythmias, but no longer for hypertension in the absence of these compelling indications.’^14^

In my view, another possible indication for beta-blockade, albeit without supporting trial data, is in those hypertensives in whom anxiety and fast heart rate help to keep up the heart rate and blood pressure, even if a tachycardia (> 100 beats/min) as strictly defined is not a prominent feature. In that case my own practice is to prefer propranolol, the only beta-blocker with anxiety as a licensed indication.

In the painful process of restoring beta-blockers to their rightful and more restricted place in cardiovascular therapy, I therefore believe my contribution, albeit modest, did help move attention away from beta-blockers as early therapy to other drugs (Table 2). Which are these other drugs? They are the angiotensin converting enzyme (ACE) inhibitors and the calcium channel blockers.

## ACE inhibitors and other renin-angiotensin system (RAS) inhibitors

**Fig. 3.**
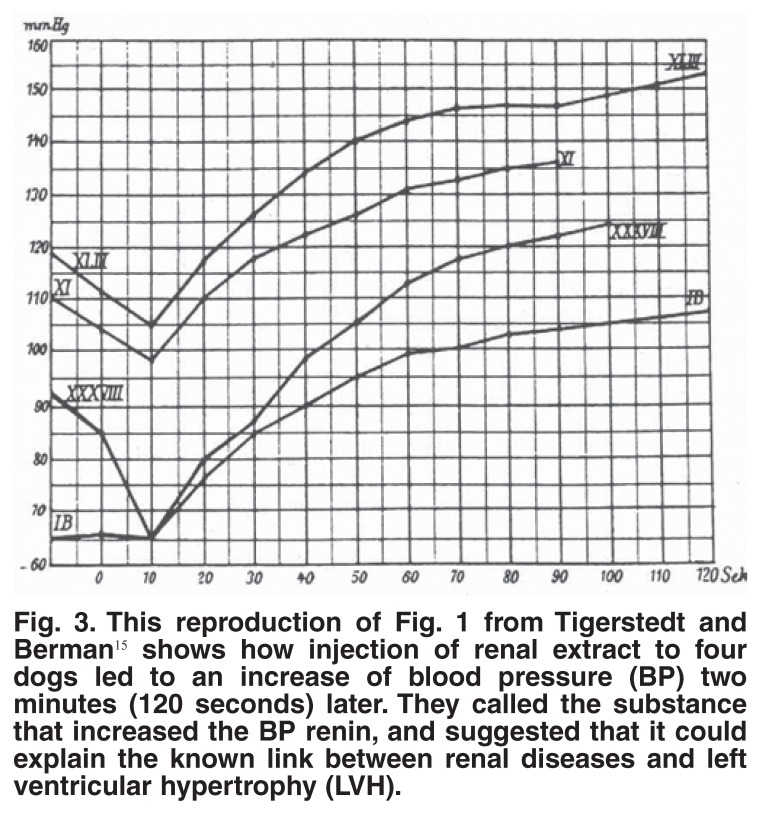
This reproduction of Fig. 1 from Tigerstedt and Berman^15^ shows how injection of renal extract to four dogs led to an increase of blood pressure (BP) two minutes (120 seconds) later. They called the substance that increased the BP renin, and suggested that it could explain the known link between renal diseases and left ventricular hypertrophy (LVH).

How did this exciting story start? It is exactly 110 years ago that Tigerstedt discovered that a renal extract increased the blood pressure of dogs and established links between the kidneys and left ventricular hypertrophy.^15^ These pioneering workers injected a renal extract into four dogs, which led to an increase in blood pressure two minutes later (Fig. 3) and, eventually, to an understanding of the links between the kidneys and left ventricular hypertrophy. It is therefore of great interest that, after so many years, the most logical therapy to inhibit the RAS would, in fact, be simple renin inhibition. Gradually the trial results are coming in with aliskiren, showing that it is simple, safe and effective (but not inexpensive).

## The advent of ACE inhibitors as antihypertensive agents

The story of the synthesis and success of the ACE inhibitor captopril was long and complex. Captopril was first clinically introduced as the antihypertensive agent most effective in renalbased hypertension. The preparatory work for the launch of captopril was the use of an intravenous angiotensin converting enzyme inhibitor, SQ20,881 which, by definition, had to be given intravenously. This brought down the blood pressure, especially in conditions of very high circulating levels of renin.^16^ The mechanism established in 1968 was competitive inhibition of the conversion of angiotensin I to angiotensin II.^17^

Publications in 1977 by Ondetti and Cushman, working at Squibb headquarters in New Jersey, USA, were coming out concurrently with clinical articles such as those by Case, Williams and Hollenberg. Captopil was also being tested in South Africa. My first clinical experience was with captopril given to an adolescent man with bilateral renal artery stenosis that was judged to be inoperable. Then already we were aware of the danger of giving captopril to such a patient, but we had no choice, as the hypertension was progressive. The representative of Squibb in South Africa flew down from Johannesburg with a supply of captopril, which we started in small amounts and gradually worked up over several days. We watched both patient and blood pressure response very carefully. In the end, the blood pressure came down and the patient survived without any renal failure.

Thereafter, we started to use captopril more and more, but in those days the recommended doses were about three to four times those presently known to be effective. Captopril-induced renal damage was a real possibility, as were loss of sensation (ageusia) and various unpleasant skin reactions. Today we know that such side effects are rare with the now-standard doses of captopril such as 25–50 mg given twice daily, or given once daily with salt restriction. Neutropenia, once conceived as a very serious, albeit uncommon side effect, has seldom been reported with these more restricted doses. Worldwide, captopril remains an important drug, one of the mainstays of ACE inhibitor therapy for hypertension and for heart failure.

## Calcium channel blockers (calcium antagonists)

Probably my major but still modest contribution to choice of antihypertensive drugs would be in the period following 1995, when Dr Curt Furberg published a highly provocative editorial in *Circulation*,^18^ suggesting that nifedipine, then only in capsule form, was increasing mortality of patients when it was given for unstable angina. I had been asked by *Circulation* to referee this article and was subsequently invited to write an editorial.^19^ The major point with which I took strong issue was that the doses used in the studies showing an increased mortality were extremely high, above 80 mg, whereas, in our practice, we were using no more than 10-mg nifedipine capsules at a time. We had already published data showing that this drug, given sublingually, was a very practical way of dealing with uncontrolled hypertension in an outpatient clinic.

Furberg was very vocal and presented his data at the European Society of Cardiology meeting in Amsterdam in August 1995. Immediately after his talk, I challenged him on some of the data because I had discovered a significant statistical error in his meta-analysis. In brief, his numbers did not add up to the totals that were given. Subsequently, Furberg started to look at the role of calcium channel blockers in hypertension and, once again, he felt that these agents had an adverse effect on mortality. Some of the meta-analyses included small, and often not very reliable studies. As time went on, it became obvious that calcium channel blockers (CCBs) were excellent antihypertensive agents and were gradually edging their way to the forefront of hypertension therapy. There were several turning points in the Furberg saga, ultimately leading to overwhelming data in favour of the CCBs and their safety, first in hypertension and, later, in effort angina.

## The hierarchy of evidence

A turning point was a conjoint article written by me, Wolfgang Kübler, then head of Cardiology at the University of Heidelberg in Germany, and Salim Yusuf, of considerable experience in designing and executing large trials that have changed our way of thinking. He educated me in the analysis of trial data, and we drew up the ‘hierarchy of evidence’, which still stands as a guide to what is reliable evidence when there are conflicting views. In a review of 100 studies, we could find no evidence that CCBs, when properly used, had unexpected adverse effects.^20^

The next major turning point was the ALLHAT study,^21^ a mega-RCT (randomised controlled trial) in which the calcium channel blocker amlodipine was compared with a diuretic and an ACE inhibitor, on outcomes in primary hypertension. The level of evidence was high. This study showed that the CCB gave the same primary-outcome results as the other agents and that there was no increase in mortality.^21^ The primary outcome in ALLHAT was fatal coronary heart disease or non-fatal myocardial infarction, combined. The four major pre-specified secondary outcomes were all-cause mortality, fatal and non-fatal stroke, combined coronary heart disease and combined cardiovascular disease (combined coronary heart disease, stroke, treated angina, heart failure and peripheral arterial disease). Individual components of the combined outcomes were pre-specified and examined. Altogether, 33 357 participants of mean age 67 years were studied for approximately 4.9 years mean duration of follow up.

The CCB and amlodipine gave exactly the same results for the primary and secondary outcome as for the other two agents. Amlodipine did, however, lead to a higher rate of heart failure than the diuretic, but the compensation was a lower rate of new diabetes with the CCB. Unexpectedly and perhaps inexplicably, the ACE inhibitor had higher rates of combined cardiovascular disease, stroke and heart failure. This trial and the ASCOT study (below) have totally laid aside the CCB’s once supposed increased mortality in hypertensive patients.

In the ASCOT blood pressure-lowering study, the comparison was between amlodipine as primary drug on one hand and the beta-blocker atenolol on the other hand, adding perindopril to the CCB as an ACE inhibitor and adding a diuretic to the atenolol as required.^22^ The ASCOT study was prematurely stopped after 5.5 years of median follow up because the all-cause mortality was reduced by 11% (*p* = 0.025) in the amlodipine–perindopril group, compared with the beta-blocker–diuretic, as well as other beneficial changes such as less new diabetes, a decreased primary endpoint and decreased total cardiovascular events and procedures. The probable mechanism for the benefit of the CCB-based regime was that it decreased the aortic pressure more than the beta-blocker-based regime for the same apparent brachial artery pressures.

It took exactly 10 years (1995–2005) to show the supremacy of CCBs as first-choice drug in the therapy of hypertension, especially when combined with an ACE inhibitor.^22^ During those 10 years, I presented many articles and talks, trying to counter the Furberg hypothesis that CCBs are harmful, but none of my contributions were nearly as important as the very positive result of the ASCOT study which, unequivocally, showed the safety of CCB-based hypertension therapy. Logically, the optimal combination is with an ACE inhibitor or, by extension, an ARB and, maybe in the future, a renin blocker.

## Evolution of the South African Hypertension Society

**Fig. 4.**
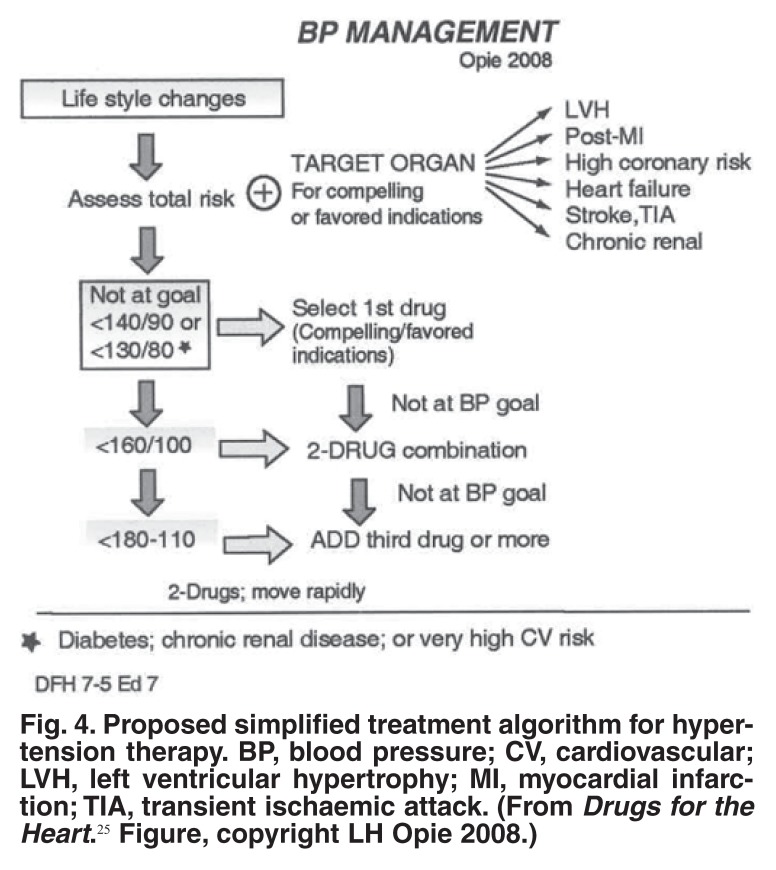
Proposed simplified treatment algorithm for hypertension therapy. BP, blood pressure; CV, cardiovascular; LVH, left ventricular hypertrophy; MI, myocardial infarction; TIA, transient ischaemic attack. (From *Drugs for the Heart.*^25^ Figure, copyright LH Opie 2008.)

Another major development has been the realisation of the crucial importance of lifestyle changes in the management of hypertension – weight loss, increased exercise and a lipid-friendly high-vegetable, high-fruit, low-salt diet. Lifestyle changes involve a complex message that has to be widely spread and emphasised and is correctly placed at the top of the therapy algorithm (Fig. 4).

Here the South African Hypertension Society has played a crucial role. Among the early founders were Profs John Milne and YK Seedat. Both have done pioneering work, with Prof Milne particularly interested in the peri-urban Johannesburg population and sustaining the Hypertension Society over many years. Prof Seedat’s interests are also wide, and include the reasons for the high rate of hypertension and diabetes in the Indian population in the Durban area. Later, I had the honour of sharing a publication with Prof Seedat on hypertension in sub-Saharan Africa. It was an excellent opportunity for me to absorb his superior knowledge, skills and judgment.^23^

I would also like to compliment Prof Krisela Steyn, who in addition to her extensive community studies, has been one of the champions of lifestyle changes, insisting that all patients with hypertension need such changes (Fig. 4). The major problem with lifestyle changes is achieving the correct motivation in any given patient. To get the message to the community is a challenge that the South African Hypertension Society is currently facing, together with the concept of a total evaluation of cardiovascular risk factors for any specific patient.

Given that for the majority of hypertensive persons in South Africa, venous blood sampling is not feasible, of great interest is the work of Dr Thomas Gaziano, from Harvard Medical School, and an affiliate of the Hatter Cardiovascular Research Institute in Cape Town, where I am currently situated. Dr Gaziano made the simple point on the basis of a large American study that, when cholesterol and lipogram measurements were not available, body mass index (BMI) was a good substitute.^24^ Therefore, by measuring the blood pressure, together with the BMI, and adding smoking history and history of diabetes, a reasonably accurate picture of the risk of a given patient with hypertension can be established, even in conditions of relatively simple clinics and day hospitals. Such estimations should now become standard in the management of hypertension.
